# Determination of antibiotic resistance genes in relation to phylogenetic background in *Escherichia coli* isolates from fecal samples of healthy pet cats in Kerman city

**Published:** 2016-12-15

**Authors:** Baharak Akhtardanesh, Reza Ghanbarpour, Sadaf Ganjalikhani, Parisa Gazanfari

**Affiliations:** 1 Department of Clinical Sciences, Faculty of Veterinary Medicine, Shahid Bahonar University of Kerman, Kerman, Iran; 2 Department of Pathobiology, Faculty of Veterinary Medicine, Shahid Bahonar University of Kerman, Kerman, Iran; 3 Graduate Student, Faculty of Veterinary Medicine, Shahid Bahonar University of Kerman, Kerman, Iran

**Keywords:** Antibiotic resistance genes, Cat, *Escherichia coli*, Phylogenetic group

## Abstract

The aim of this study was to determine antibiotic resistance genes, phylogenetic groups and anti-microbial resistance patterns of *Escherichia coli* isolates from fecal samples of healthy pet cats in Kerman city. Ninety* E. coli* isolates were recovered from obtained rectal swabs. Antibiotic resistance pattern of the isolates against seven selected antibiotic was determined using disc diffusion method. Phylogenetic background of the isolates was determined according to the presence of the *chuA*, *yjaA* and *TspE4C2* markers. Theisolates were examined to determine a selection of antibiotic resistance genes including *tetA, tetB, aadA, sulI *and* dhfrV *by polymerase chain reaction. Forty two isolates (46.6%) were positive at least for one of the examined genes. Phylotyping revealed that the isolates are segregated in phylogenetic groups A (66.7%), B1 (1.2%), B2 (13.4%) and D (18.9%). Among 90 isolates, 26.6% were positive for *tetB *gene, 10.0% for c*qnrS* gene, 12.3% for *sulI* and *aadA* genes, 8.9% for *tetA* and 2.2% for *dhfrV*gene. None of the *E. coli *isolates were positive for *qnrA* and *qnrB* genes. Sixteen combination patterns of antibiotic resistance genes were identified which belonged to four phylogroups. Maximum and minimum resistant isolates were recorded against to tetracycline (82.3%) and gentamycin (1.2%), respectively. Fifteen antibiotic resistance patterns were determined in different phylo-genetic groups. In conclusion, feces of healthy pet cat in Kerman could be a source of antibiotic resistant *E. coli* isolates, whereas these isolates were distributed all over the main phylogroups.

## Introduction

*Escherichia* *coli *isolates are commonly found in the gastrointestinal tract of animals and humans, and can also be implicated in infectious diseases.^1^ Based on genetic and clinical manifestations, *E. coli* isolates are classified into intestinal pathogenic, extra-intestinal pathogenic and commensal groups. ^2^ Although extra-intestinal pathogenic *E.coli* isolates are pathogens involved in several disease conditions, ranging from urinary tract infection to meningitis in humans and animals, the emergence of antibacterial resistant strains in both commensal and pathogenic bacteria has become an important public health issue.^3^ Resistance to various antibiotic classes is widespread in *E. coli *isolated from animals and humans and may compromise treatment efficacy since fluoroquinolones, aminoglycosides, sulfonamide and cephalosporins anti-microbials are frequently used to treat Gram-negative infections.^4^ Companion animals (cats and dogs) represent potential sources of distribution of antimicrobial resistance because of the widespread use of antimicrobial agents in these animals and their close contact with humans.^5^ Alarming state of multidrug-resistant (MDR) is seen in examining the *Escherichia coli* associated with infections in cats and dogs throughout the United States and Europe.^5^^,^^6^ Selection of proper antimicrobials is crucial for effective therapy of extra-intestinal and gastrointestinal infections and substantially decreases the risk of development of MDR in commensal or pathogenic bacteria.^7^ Several fluoroquinolones have been approved for treatment of bacterial infections in cats. Thereby, increasing resistance to fluoroquinolones in *E. coli* has been observed throughout the world. Treatment can be complicated when *E. coli* isolates resistant to fluoroquinolones exhibit MDR pheno-types.^8^ Tetracycline resistance has been linked to prolonged use in animals. The tetracycline resistance genes identified in studies of *E. coli* isolates are *tetA*,* tetB*,* tetC *and *tetD*.^9^ Resistance to aminoglycosides is mediated in *E. coli* by genes from N-acetyltransferases, O-adenyl-transferases and O-phosphotransferases classes of aminoglycoside modifying enzymes.^10^ Resistance to the sulfonamides can be conferred by resistance genes,* sul1*,* sul2*, and *sul3*. Several mechanisms of resistance to trimethoprim have been identified.^9^^, ^^11^

*Escherichia coli *strains can be classified to one of the four phylogenetic groups: A, B1, B2 or D based on the presence or absence of *chuA*, *yjaA* genes and an anonymous DNA fragment, *TspE4C2* by triplex polymerase chain reaction (PCR). The commensal *E. coli *strains are belonged to groups A and B1, whilst the antibiotic resistances of *E. coli* strains usually belong to non-B2 phylogenetic group shift towards group A.^12^^,^^13^

There are few reports on the prevalence and importance of antibiotic resistance genes and phylogenetic groups of *E. coli* isolates from healthy pet cats. The objectives of this study were to determine the antibiotic resistance genes and antimicrobial resistance patterns of *E. coli* isolates from fecal samples of healthy pet cats by phenotypic (disk diffusion) and genotypic (i.e. PCR) methods and characterization of the isolates according to their phylogenetic background in Southeastern Iran, Kerman.

## Materials and Methods

Bacteriological examination. Rectal swab samples were obtained from 90 healthy pet cats. All of the sampled animals were admitted in the small animal clinic of teaching veterinary hospital of Shahid Bahonar university of Kerman, from November 2013 to September 2014. A detailed questionnaire was completed for each animal to get information about age, sex, lifestyle and clinical histories. Swab samples were streaked onto Mac Conkey agar (Biolife Laboratories, Milan, Italy) and the plates were incubated at 37 ˚C for 24 hr for isolation of E. coli in the single colonies. The presumptive E. coli colonies were further streaked onto eosin methylene blue and incubated overnight at 37 ˚C again. Green metallic sheen colonies indicative of E. coli were subjected to biochemical tests, including indole, methyl red, Voges-Proskauer and Simmons citrate tests for E. coli identification. The isolates were confirmed to be E. coli using biochemical API 20E identification system (BioMe´rieux, Marcy l'Etoile, France). Then, the confirmed isolates were stored in Luria-Bertani broth (Invitrogen, Paisley, Scotland) with 30% sterile glycerol at – 80 ˚C for molecular studies. From each sample one confirmed isolate was chosen for PCR assays and antibiotic susceptibility tests.

PCR assays for antibiotic resistance genes. Freshly grown over night cultures on Luria-Bertani agar (Merck, Darmstadt, Germany) of E. coli isolates and reference strains were used for DNA extraction by lysis method.^14^ Specific primers (TAG Copenhagen, Frederiks-berg, Denmark) used for amplification of the genes are presented in Table 1. Presence of quinolone-resistance encoding genes qnrA, qnrB and qnrS were assayed as described by Cattoir et al ^15^_. _Five μL of extracted DNA from each isolates were subjected to multiplex PCR in a 50 μL reaction mixture. Amplification was carried out as follow: 10 min at 95 ˚C and 35 cycles of amplification consisting of 1 min at 95 ˚C, 1 min at 54 ˚C and 1 min at 72 ˚C and 10 min at 72 ˚C for the final extension.

Detection of tetracycline resistance genes tetA and tetB was performed using uniplex PCR assays.^16^ The PCR was performed in a total volume of 50 μL containing 5 μL of the extracted DNA with final concentration of 1.5 mM MgCl_2_, 2.5 μMof each dNTP (Fermentas, Vilnius, Lithuania), 0.5 μL of each primer pair and 1 U of Taq polymerase (Fermentas). The PCR amplification was conducted in MJ mini personal thermal cycler (Bio-Rad Laboratories, Hercules, USA). The uniplex PCR amplification conditions consisted of initial denaturation at 94 ˚C for 5 min, with 30 cycles of denaturation at 94 ˚C for 30 sec, annealing at 50 ˚C for 30 sec, extension at 72 ˚C for 1 min and final cycle of amplification at 72 ˚C for 10 min.

Three antibiotic resistance genes sulI (sulfonamide), dhfrV (trimethoprim) and aadA (aminoglycoside) were determined using a multiplex as described previously.^16^ The PCR amplification was conducted with the following conditions: Initial denaturation at 95 ˚C for 15 min,  followed by 30 cycles of denaturation at 94 ˚C for 30 sec, annealing at 58 ˚C for 30 sec, and extension at 72 ˚C for 1 min and final cycle of amplification at 72 ˚C for 10 min. The PCR products were visualized by electrophoresis at 80 V, 500 mA in 1.5% agarose gels prepared in 0.5X tris-borate-EDTA buffer (EURx Ltd., Gdańsk, Poland).

**Table 1:** The specific primers used in this study. Expected size of products and references used for amplification conditions of target genes were presented

**Gene **		**Primer Sequence (5′-3′)**	**Product size (bp)**	**Reference No**
***tetA***	**Tetracycline**	GTGAAACCCAACATACCCC GAAGGCAAGCAGGATGTAG	877	16
***tetB***		CCTTATCATGCCAGTCTTGC ACTGCCGTTTTTTCGCC	773	16
***qnrA***	**Quinolone**	AGAGGATTTCTCACGCCAGG TGCCAGGCACAGATCTTGAC	580	15
***qnrB ***		GGMATHGAAATTCGCCACTG TTTGCYGYYCGCCAGTCGAA	264	15
***qnrS***		GCAAGTTCATTGAACAGGGT TCTAAACCGTCGAGTTCGGCG	428	15
***aadA***	**Aminoglycoside**	TGATTTGCTGGTTACGGTGAC CGCTATGTTCTCTTGCTTTTG	284	16
***sulI***	**Sulfonamide**	TTCGGCATTCTGAATCTCAC ATGATCTAACCCTCGGTCTC	822	16
***dhfrV***	**Trimethoprim **	CTGCAAAAGCGAAAAACGG AGCAATAGTTAATGTTTGAGCTAAAG	432	16
***chuA***	**Phylogenetic group**	GACGAACCAACGGTCAGGAT TGCCGCCAGTACCAAAGACA	279	12
***yjaA***		TGAAGTGTCAGGAGACGCTG ATGGAGAATGCGTTCCTCAAC	211	12
***TspE4C2***		GAGTAATGTCGGGGCATTCA CGCGCCAACAAAGTATTACG	152	12

Phylogenetic grouping. The phylogenetic groups of isolates were determined using a triplex PCR and the isolates were classified as A, B1, B2 and D phylo-groups.^12^^,^^17^ The combinations of three genetic markers (chuA, yjaA and TSPE4C2) were used to determine four phylogenetic groups as follow: ChuA^-^, YjaA^-/+^ and TSPE4C2^-^ was assigned to group A, ChuA^-^, YjaA^-/+^ and TSPE4C2^+^ was assigned to group B1, chuA^+^, YjaA^+^ and TSPE4C2^-/+^ was assigned to group B2 and ChuA+, YjaA^-^ and TSPE4C2^-/+^ was assigned to group D.^14^

Antibiotic susceptibility test. Antibiotic resistance profile of isolates against seven selected antibacterial agents was determined by disc diffusion method according to Clinical and Laboratory Standards Institute's (CLSI) guidelines.^18^ The following antimicrobial discs (Padtan Teb, Tehran, Iran) were used in disc diffusion assay: trimethoprim/sulfamethoxazole (SXT; 25 μg), tetracycline (TE; 30 µg), gentamycin (GM; 10 µg), kanamycin (K; 30 µg), enrofloxacin (NFX; 5 μg), streptomycin (S; 10 µg) and florfenicol (FF; 30 µg). The E. coli strain ATCC 25922 was served as a control in all assays.

## Results

Bacteriological examination. In bacteriological examinations, 90 E. coli were isolated from the same number of rectal swab samples of healthy pet cats.

**Fig. 1:**
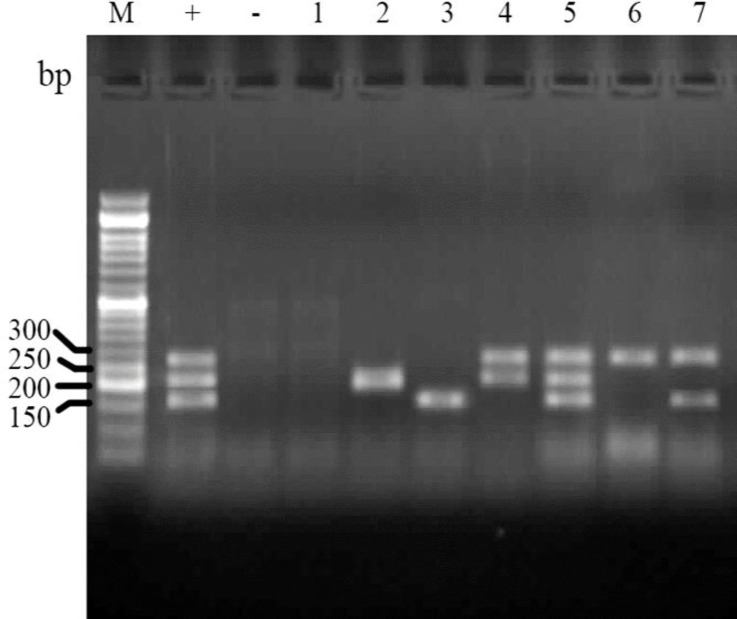
Electrophoresed gel image of PCR products related to detection of phylogenetic groups. Lane M: Ladder (50 bp). Lane +: Positive control (chuA, yjaA and TspE4C2), Lane -: Negative control, Lane 1: A (chuA-, yjaA- and TspE4C2- template, Lane 2: A (yjaA+) template, Lane 3: B1 (TspE4C2+) template, Lane 4:B2 (yjaA+, chuA+) template, Lane 5: B2 (chuA+, yjaA+ and TspE4C2+), Lane 6: D (chuA+) template, Lane 7: D (chuA+, TspE4C2+) template

Phylogenetic grouping of isolates. Analysis of PCR results for determination of phylogenetic groups showed that the E. coli isolates are belonged to four main groups of A (66.7%), B1 (1.2%), B2 (13.4%) and D (18.9%) phylo-groups (Fig. 1).

Detection of antibiotic resistance genes by PCR. Multiplex and simplex PCR tests results showed that 46.7% of isolates (42) are positive at least for one of the examined antibiotic resistance genes, whereas 48 isolates (53.3%) are negative for these genes. Among 90 isolates examined for presence of eight antibiotic resistance genes, tetracycline resistance encoding gene tetB was the most prevalent resistance gene. None of the isolates was positive for qnrA and qnrB genes.

**Fig. 2:**
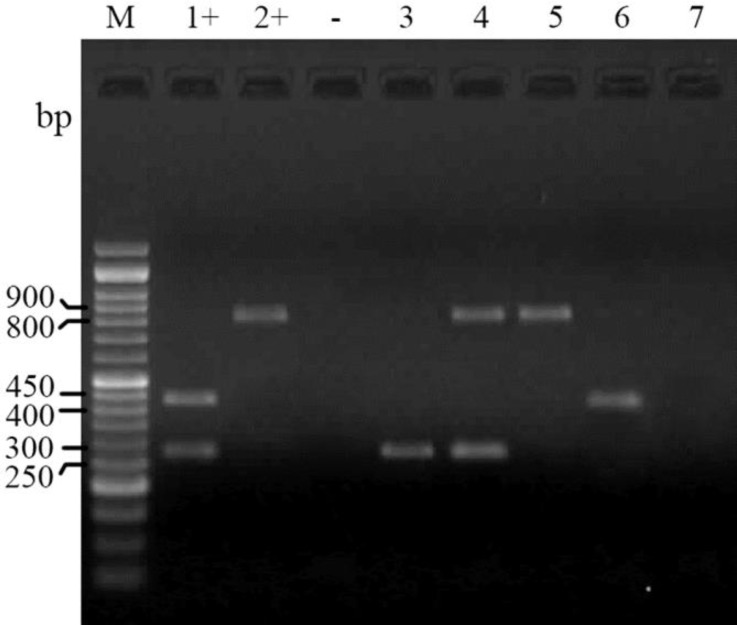
Electrophoresed gel image of PCR products related to detection of antimicrobial resistance aadA, sulI and dhfrV genes. Lane M: Ladder (50 bp), Lane 1+: Positive control (aadA and dhfrV), Lane 2+: Positive control (sulI), Lane -: Negative control, Lane 3: aadA template, Lane 4: sulI and aadA template, Lane 5: sulI template, Lane 6: dhfrV template,  Lane 7: Negative template

The PCR tests for determination of antibiotic resistance encoding genes for aminoglycoside (aadA), sulfonamide (sulI) andtrimethoprim (dhfrV) revealed that 12.3% (11) of isolates are positive for sulI and aadA genes and 2.2% (2) of isolates were positive for dhfrV gene (Fig. 2).

**Fig. 3:**
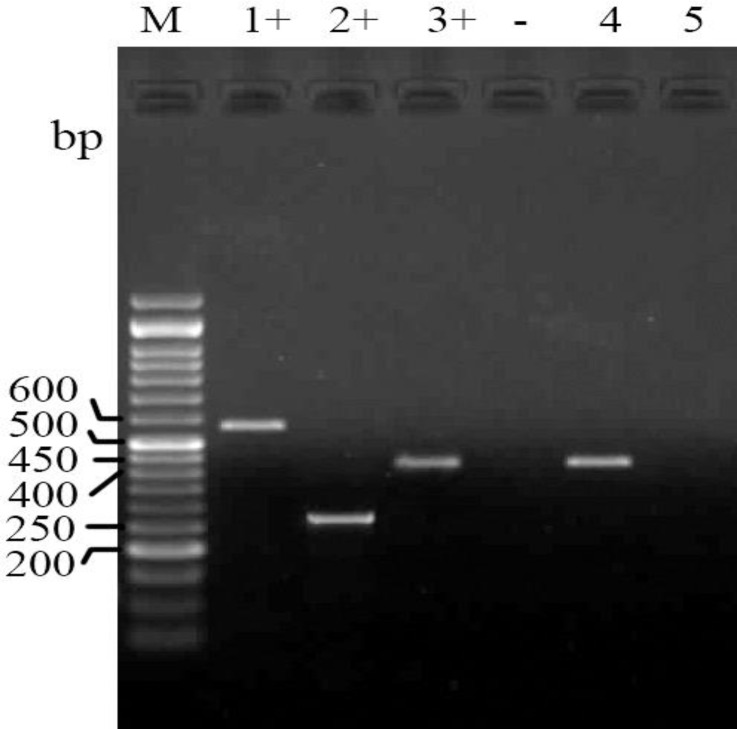
Electrophoresed gel image of PCR products related to detection of antimicrobial resistance qnrA, qnrB and qnrS genes. Lane M: ladder (50 bp), Lane 1+: Positive control (qnrA), Lane 2+: Positive control (qnrB), Lane 3+: (qnrS) template, Lane -: Negative control, Lane 4: qnrS. template, Lane 5: Negative template

The PCR results for detection of quinolone resistance encoding genes qnrA, qnrB and qnrS showed that only nine isolates (10.0%) contain the qnrS gene (Fig. 3).

**Fig. 4:**
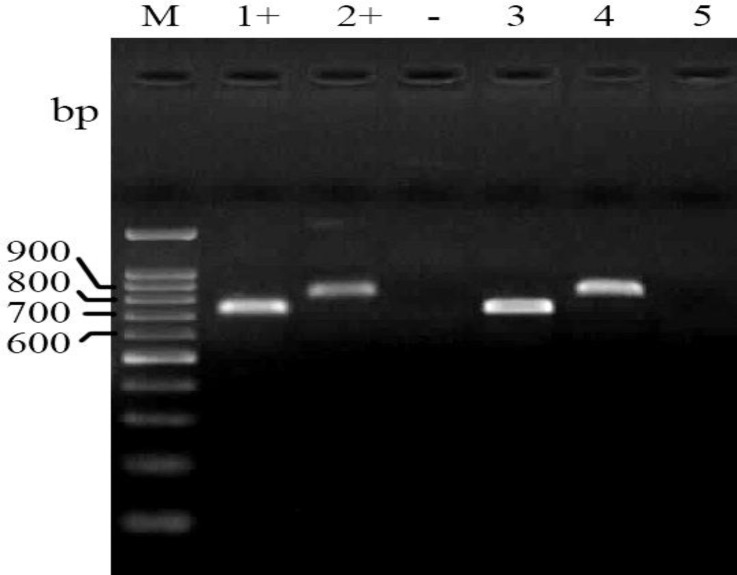
Electrophoresed gel image of PCR products related to detection of antimicrobial resistance tetAand tetB genes. Lane M: Ladder (100 bp), Lane 1+: Positive control (tetB), Lane 2+: Positive control (tetA), Lane -: Negative control, Lane 3: tetB template, Lane 4: tetA template, Lane 5: Negative template

The PCR assays for detection of tetA and tetB showed that 8.9% (8) and 26.6% (24) of isolates are positive, respectively (Fig. 4).

Phylogenetic background of positive isolates for antibiotic resistance genes. Forty four positive isolates for antibiotic resistance genes were distributed in four phylogenetic groups A (27), B1 (1), B2 (5) and D (11). The PCR assays revealed that 11 sulI positive isolates are belonged toA (10) and B2 (1) phylogroups. Eleven aadA positive isolates were distributed in A (8), B1 (1), B2 (1) and D (1) phylogenetic groups whereas both of dhfrV positive isolates are belonged to A phylo-group. Phylogenetic assay showed that 10 qnrS isolates are segregated in A (3), B1 (1), B2 (2) and D (4) phylogroups. Analyses of PCR results showed that 8 tetA positive isolates were belonged to A (7) and B2 (1) phylogroups whereas tetB positive isolates fell into three phylogenetic groups A, B2 and D. The positive isolates for a combination of antibiotic resistance genes were distributed in all of phylogenetic groups (Table 2).

**Table 2:** Distribution of positive isolates for antibiotic resistance genes in relation to phylogenetic groups

**Combination patterns of antibiotic resistance genes**	**Phylogenetic group**				**Total**
	**A**	**B1**	**B2**	**D**	
***tetB***	7	-	3	4	14
***tetA***	4	-	-	2	6
***dhfrV***	2	-	-	-	2
***sulI***	1	-	-	-	1
***qnrS***	1	-	-	2	3
***aadA***	-	-	-	1	1
***aadA, tetA***	2	-	-	-	2
***qnrS, tetB***	1	-	1	2	4
***qnrS, sulI***	1	-	-	-	1
***aadA, sulI***	1	-	-	-	1
***sulI, tetA***	1	-	-	-	1
***aadA, tetB***	1	-	-	-	1
***sulI, tetB***	1	-	-	-	1
***aadA, sulI, tetA***	-	-	1	-	1
***aadA, qnrS, aadA***	-	1	-	-	1
***aadA, sulI, tetB***	4	-	-	-	4
**Total**	27	1	5	11	44

**Table 3:** Phylogenetic groups of antibiotic resistant isolates according to disk diffusion test

**Antibiotic resistance patterns**	**Phylogenetic group**				**Total**
	**A**	**B1**	**B2**	**D**	
**TE**	29	-	9	7	45
**TE, S**	1	-	-	-	1
**TE, K**	2	-	-	-	2
**FF, TE**	3	-	-	-	3
**NFX, TE**	2	-	-	-	2
**SXT, TE**	1	-	-	-	1
**FF, K ,TE**	1	-	-	-	1
**NFX, SXT, TE**	2	-	-	-	2
**FF, K, SXT, TE**	1	-	-	-	1
**FF, NFX, S, TE**	1	-	-	-	1
**NFX, K, SXT, TE**	2	-	-	-	2
**FF, S, SXT, TE**	-	-	-	1	1
**FF, GM, NFX, TE**	1	-	-	-	1
**FF, NFX, SXT, TE**	3	-	-	-	3
**FF, NFX, SXT, K , S, TE **	5	1	1	1	8
**Total**	54	1	10	9	74

**SXT:** Trimethoprim/Sulfamethoxazole, **TE:** Tetracycline, **GM:** Gentamycin, **K:** Kanamycin, **NFX:** Enrofloxacin, **S: **Streptomycin, **FF:** Florfenicol.

## Discussion

 Emergence of antimicrobial resistance is a serious problem in veterinary medicine.^19^ Development and persistence of antibiotic resistance in commensal and non-pathogenic bacteria are among worldwide concerns, because they are thought to act as reservoirs of resistance genes capable of transferring genes to foodborne and other zoonotic pathogens.^20^ This may cause difficulty in treating animal infections in the future and merits further analyses.^7^ The most frequent causes of antimicrobial treatments in small animals such as cats and dogs are wound wound, respiratory and urinary tract infections and otitis externa. Uropathogenic E. coli strains are the most important infectious causes of urinary tract diseases in dogs, cats and human.^21^

One of the purposes of the current study was to determine the prevalence of resistant isolates by phenotypic and genotypic tests. In the present study, high prevalence of tetracycline resistant isolates was observed. Previous study on E. coli isolates from clinical specimens of cats and dogs during seven years revealed a significant increase in frequency of resistant to tetracycline from 45.3% in 2007 to 74.4%.^7^ Tetracyclines have been used in animal and human medicine and resistance genes are easily acquired.^16^ In the present study, phylogenetic analysis of tetracycline resistant isolates by phenotypic and genotypic tests confirmed that isolates fell into four phylogenetic groups. There are few reports about phylo-genetic background of tetracycline resistant E. coli isolates from pet animals. The results of present study showed 10.0% of examined samples are resistant against quinolone according to genotypic examinations and are distributed in four phylogenetic groups. Fluoroquinolones are broad-spectrum antimicrobial drugs that are highly effective for the treatment of various animal infections.^4^ Presence of plasmid-mediated quinolone resistance has been reported in phenotypically ESBL-producing E. coli isolates from feline and canine clinical samples in USA. The qnrA and qnrB genes were not detected, which is similar to the results of the current study.^8^

A study on E. coli isolates from dogs with urinary tract infections showed that fluoroquinolone-resistant isolates differ significantly from fluoroquinolone susceptible isolates based on phylogenetic group distribution, with fluoroquinolone-resistant isolates being comparatively enriched for groups A and B1 and fluoroquinolone-susceptible isolates comparatively enriched for group B2.^22^

In the current study, approximately 12.0% of the isolates were positive for sulI gene, whereas lower prevalence of resistant E. coli was observed in dhfrV gene (2.3%). Determination of sulfonamide resistance encoding genes helps understanding the mobilization capability of sul genes among different bacterial species and resistance disseminates in various sources.^23^ Increased resistance to trimethoprim-sulfamethoxazole in feline and canine E. coli isolates may have been resulted from selection pressure or different exposure to resistant E. coli in the environment.^24^ In the present study, most of positive isolates for sulI gene were belonged to phylo-group A. In Switzerland 37.0% of E. coli isolates from humans and animals (dogs, cats, swine, cattle and poultry) carried a class 1 integron and 34.2% of the isolates also carried the sulI gene. The positive E. coli isolates for integrons were more prevalent in the phylogenetic group A.^25^

Phylogenetic analyses showed that E. coli isolates from healthy cats fell into four main phylogenetic groups (A, B1, B2 and D), which most antibiotic resistance genes positive isolates fell into A and D groups. In human, uropathogenic E. coli isolates’ antimicrobial resistance is often associated with reduced virulence and shifts toward non-B2 phylo-genetic groups.^26^ It is noteworthy that the most commensal and diarrheagenic strains belong to group A, while virulent extra-intestinal strains belong mainly to groups B2 and, to a lesser extent, D. In addition, in phylogenetic groups B2 and D, the percentage of antibiotic resistance genes positive strains is significantly lower.^12^^,^^13^ In a study, 69.0% of ESBL-producing E. coli isolates from clinical specimens of cats and dogs E. coli isolates were belonged to phylogenetic group D.^10^ It is reported that multidrug-resistant E. coli isolates from nosocomial infections in hospitalized dogs are belonged to phylogenetic groups A and D.^27^ In the current study, to investigate the relationships between genetic background and antibiotic resistance profile, the phylogenetic distribution of antibiotic resistant isolates was determined pheno-typically. According to the results of phenotypic tests, maximum and minimum prevalences of resistance were observed against tetracycline and gentamycin, respectively.

In conclusion, moderate to high levels of resistance against antibacterial agents have been detected in E. coli isolates from healthy pet cats in Kerman city. In general, these levels of resistance were lower than those previously reported for farm animals and poultry isolates, which might be result of a low antibacterial pressure in the cats in comparison with the farm animals and poultry. Cats and dogs are companion animals that are in close contact with humans since ancient times and antimicrobials resistance appears to be increasing in commensal bacterial isolates as well as pathogens.The emergence and spread of antimicrobial resistance are major public health concerns of the world. Indicator commensal bacteria, such as E. coli, are useful for the survey of antimicrobial resistance. Further studies need to be conducted using molecular-epidemiological assays and detailed analyses of the data to determine the association between antibiotic resistance and phylogenetic background of E. coli isolates from healthy cats. The results of this study demonstrated that E. coli isolates from healthy cats belonging to different phylo-groups contain several antibiotic resistance genes in combination to each other. Further studies should be carried out to identify the importance of antibiotic resistances in companion animals in relation to phylo-genetic background of E. coli isolates.
